# Left Internal Carotid Artery Arising from the Right Cavernous Internal Carotid Artery: A Case Report

**DOI:** 10.7759/cureus.1807

**Published:** 2017-10-27

**Authors:** Charlotte Wilson, Bill H Wang, Joe Iwanaga, Akil Patel, Josh Bentley, R. Shane Tubbs, Akshal S Patel

**Affiliations:** 1 Seattle Science Foundation; 2 Swedish Neuroscience Institute; 3 Neurosurgery, Advocate Health Care; 4 Neurosurgery, Seattle Science Foundation; 5 Neurosurgery, Swedish Medical Center

**Keywords:** internal carotid artery, agenesis, abnormality, collateral circulation, anastomosis

## Abstract

Anatomical variations involving the internal carotid artery are uncommon. Herein, we present a very rare origin of the internal carotid artery. An adult female presented to the emergency department after falling. Imaging revealed that the left internal carotid artery arose from the contralateral cavernous segment of the internal carotid artery. Such a variation should be kept in mind by radiologists and surgeons who interpret and operate in this area, respectively.

## Introduction

The internal carotid artery originates from the carotid bifurcation and enters the cranium through the carotid canal located in the petrous part of the temporal bone [1]. It passes anteromedially and then superomedially above the cartilage of the foramen lacerum. Within the cavernous sinus, the internal carotid artery turns anteriorly to the side of the body of the sphenoid bone, continuing to curve superiorly and medial to the anterior clinoid process to pierce the dural roof of the sinus [1].

Agenesis, aplasia, or hypoplasia of the internal carotid artery is extremely rare with an incidence of <0.01% [2-3]. Midkiff et al. defined the agenesis of the internal carotid artery as the absence of the carotid canal as well as the cervical and petrous portions of the internal carotid artery [4]. Aplasia refers to the presence of the carotid canal as well as primitive remnants of the vessel [4]. Hypoplasia has been defined as an underdeveloped internal carotid artery in conjunction with a small, bony carotid canal [5]. Agenesis of the internal carotid artery is more frequently observed in the left internal carotid artery and bilateral agenesis appears in less than 10% of the cases of internal carotid artery agenesis [6]. In this report, we present a very rare case of the internal carotid artery arising from the cavernous section of the contralateral internal carotid artery and lacking its cervical and petrous portions.

## Case presentation

**Figure 1:**
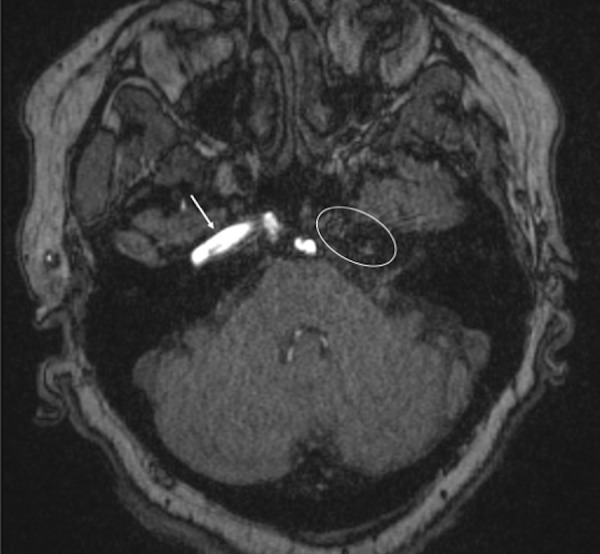
Axial 3D time of flight MRI of the head, noting absence of the carotid canal and internal carotid artery at the skull base (oval). For comparison, note the right-sided internal carotid artery (arrow) at the skull base.

**Figure 2:**
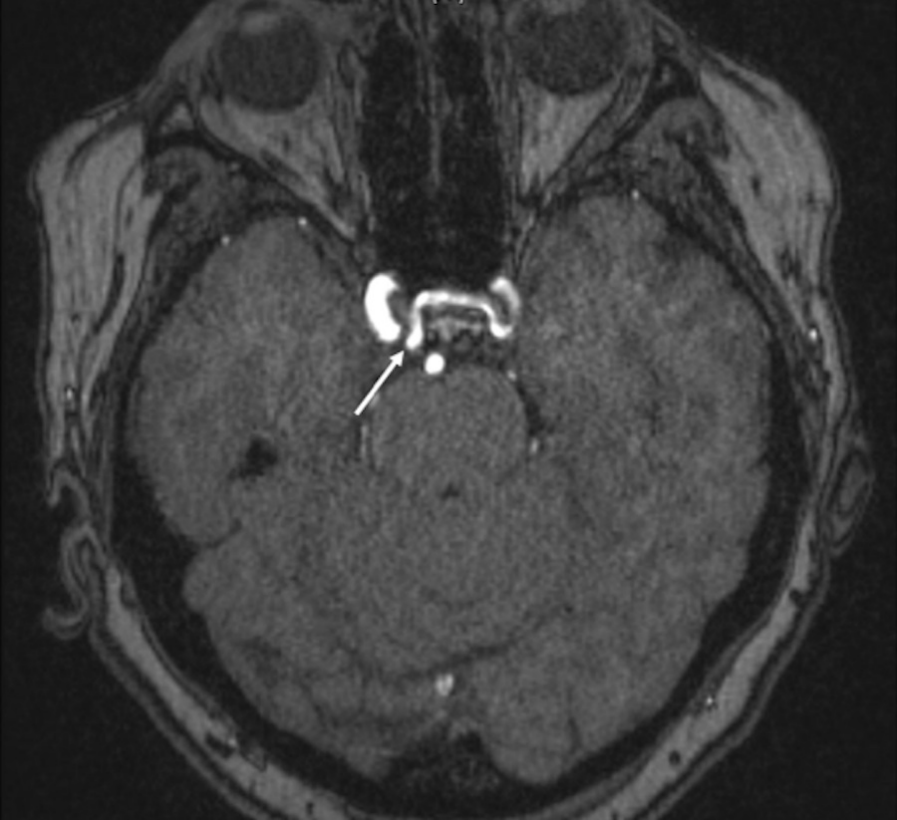
Axial 3D time of flight MRI of the head, noting the unusual origin of the left internal carotid artery (arrow) from the contralateral cavernous segment of the right internal carotid artery. Note the course of the left internal carotid artery through the floor of the sella turcica.

**Figure 3:**
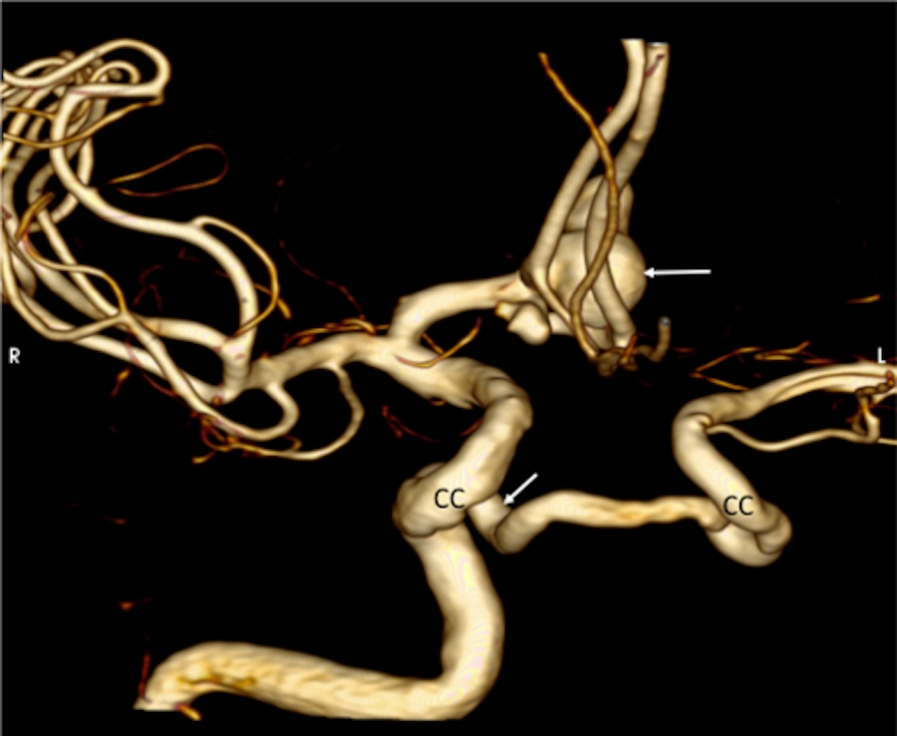
3D reconstruction from a CT angiography, noting the anterior circulation aneurysm (upper arrow) and the origin of the left internal carotid artery from the right cavernous segment (CC) of the right internal carotid artery (lower arrow).

A 59-year-old white female presented to the emergency room after slipping and falling on ice. She had a past medical history of hypertension, hyperlipidemia, and asthma. The patient smoked one half pack of cigarettes per day. An axial 3D time of flight magnetic resonance imaging (MRI) scan was performed and identified the absence of the left carotid canal with an absence of flow signal (Figure 1). An unusual vessel was also seen to arise from the right cavernous internal carotid artery (Figure 2). The vessel traveled in the floor of the sella turcica (Figure 2). Additional imaging with 3D reconstruction from a computed tomography (CT) angiography better defined an aberrant origin of the left internal carotid artery (Figure 3). CT angiography noted the presence of an anterior communicating artery aneurysm, which was bilobed and measured 7 x 11 mm. The physical examination was unremarkable for any neurological deficits. Angiography found that the aneurysm was not amenable to endovascular treatment. Therefore, the patient underwent a right-sided craniotomy for clipping of her complex aneurysm and was soon discharged home with no complications.

## Discussion

The internal carotid artery first appears at the 3 mm embryonic stage, originating from the dorsal aorta and the third aortic arch, with full development by the sixth week [7-8]. The carotid canal begins forming between the fifth and sixth week of development, and its presence is dependent on the successful formation of the internal carotid artery [7]. Huber postulated that defective regression of the third pharyngeal arch artery might result in an anatomy similar to our current case [9].

**Figure 4:**
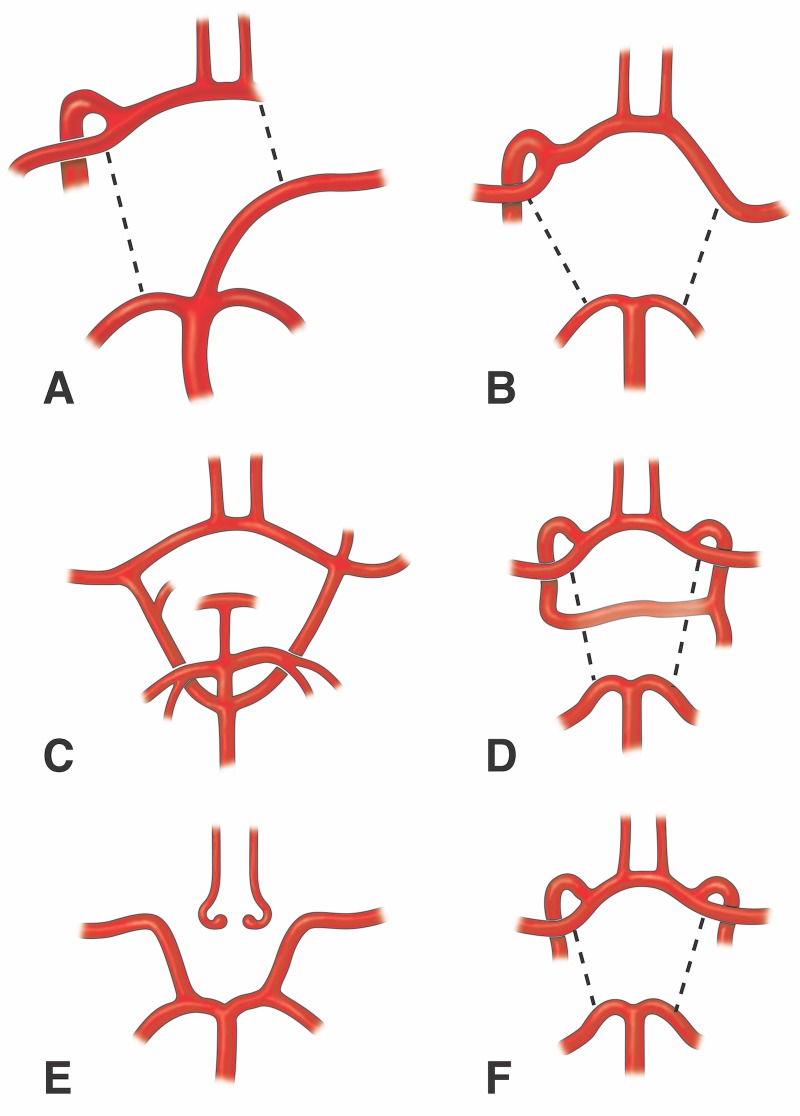
Schematic drawings of the classification system for carotid artery variants used by Lie. A. Fetal anastomoses used in cases of left internal carotid artery absence. B. Adult type of A classification. C. Agenesis of both internal carotid arteries. D. Variant of the case reported herein. E. Hypoplasia of the left and right internal carotid arteries. F. Bilateral aplasia of the cervical segments of the internal carotid arteries.

Variants of the internal carotid artery are uncommon [10]. Unilateral agenesis of the internal carotid artery is a rare anomaly that usually presents asymptomatically with few patients presenting headaches, subarachnoid hemorrhage, or transient ischemic attack [5]. This is due to adequate cerebral circulation from the circle of Willis, intercavernous and external carotid artery anastomoses, and/or the remaining primitive embryonal arteries. Collateral blood flow in the case of internal carotid artery agenesis is reliant on the stage in which the disruption in development occurred [8]. The circle of Willis is formed between the 7 and 24 mm embryotic stage. If a disruption occurs before the completion of the circle of Willis, primitive pathways of collateral circulation will dominate. If the disruption occurs after the 24 mm embryonic stage, the circle of Willis will succeed due to its developmental completion. Lie classified (Figure 4) six primitive pathways of collateral blood flow in the absence of an internal carotid artery, with type D representing an intercavernous communication [10].

Previous reports of an internal carotid artery with intercavernous anastomosis (transverse carotid anastomosis) are rare. Midkiff et al. presented a case of agenesis of the internal carotid artery agenesis with an intercavernous anastomosis [4]. Injection to the left carotid artery revealed a collateral vessel uniting the cavernous sections of the internal carotid arteries [4]. Lie also described similar findings [10]. A left carotid arteriogram showed an anomalous vessel located near the clivus connecting the carotid siphons of the existing internal carotid artery and the aplastic internal carotid artery [10]. Lie speculated that the origin of such an anosmatic vessel was due to the fusion of two primitive trigeminal arteries that failed to establish their typical connections to the basilar artery [10]. Tasar et al. identified a similar case in a review of over 5,000 angiograms [6]. Based on our review of the literature and an examination of our case, the term intercavernous anastomosis is an inappropriate term to describe this anomaly and should be replaced with a contralateral cavernous origin of the internal carotid artery. Such an anatomical variation is similar to a contralateral origin of the anterior cerebral artery that is seen much more commonly.

## Conclusions

Based on our report and a review of the literature, the origin of the internal carotid artery from the contralateral cavernous segment of the internal carotid artery is very rare. This anatomical variant should not be considered an intercavernous anastomosis but rather a true origin. Clinicians should be aware that such a variation exists, especially with invasive procedures near the sella turcica (e.g., transnasal approaches to the pituitary gland), as an injury to this anomaly could have catastrophic consequences.
